# Unique active-site and subsite features in the arabinogalactan-degrading GH43 exo-β-1,3-galactanase from *Phanerochaete chrysosporium*

**DOI:** 10.1074/jbc.RA120.016149

**Published:** 2021-01-13

**Authors:** Kaori Matsuyama, Naomi Kishine, Zui Fujimoto, Naoki Sunagawa, Toshihisa Kotake, Yoichi Tsumuraya, Masahiro Samejima, Kiyohiko Igarashi, Satoshi Kaneko

**Affiliations:** 1 Department of Biomaterial Sciences, Graduate School of Agricultural and Life Sciences, University of Tokyo, Tokyo, Japan; 2 Advanced Analysis Center, National Agriculture and Food Research Organization (NARO), Tsukuba, Ibaraki, Japan; 3 Department of Biochemistry and Molecular Biology, Faculty of Science, Saitama University, Saitama, Japan; 4 Faculty of Engineering, Shinshu University, Nagano, Japan; 5 VTT Technical Research Centre of Finland, Espoo, Finland; 6 Department of Subtropical Bioscience and Biotechnology, Faculty of Agriculture, University of the Ryukyus, Nishihara, Okinawa, Japan

**Keywords:** glycoside hydrolase family 43, carbohydrate-binding module family 35 exo-β-13-galactanase, arabinogalactan-protein, Phanerochaete chrysosporium, enzyme structure, carbohydrate metabolism, carbohydrate-binding protein, plant cell wall, biodegradation, carbohydrate-binding module family 35, exo-β-1,3-galactanase

## Abstract

Arabinogalactan proteins (AGPs) are plant proteoglycans with functions in growth and development. However, these functions are largely unexplored, mainly because of the complexity of the sugar moieties. These carbohydrate sequences are generally analyzed with the aid of glycoside hydrolases. The exo-β-1,3-galactanase is a glycoside hydrolase from the basidiomycete *Phanerochaete chrysosporium* (*Pc*1,3Gal43A), which specifically cleaves AGPs. However, its structure is not known in relation to its mechanism bypassing side chains. In this study, we solved the apo and liganded structures of *Pc*1,3Gal43A, which reveal a glycoside hydrolase family 43 subfamily 24 (GH43_sub24) catalytic domain together with a carbohydrate-binding module family 35 (CBM35) binding domain. GH43_sub24 is known to lack the catalytic base Asp conserved among other GH43 subfamilies. Our structure in combination with kinetic analyses reveals that the tautomerized imidic acid group of Gln^263^ serves as the catalytic base residue instead. *Pc*1,3Gal43A has three subsites that continue from the bottom of the catalytic pocket to the solvent. Subsite −1 contains a space that can accommodate the C-6 methylol of Gal, enabling the enzyme to bypass the β-1,6–linked galactan side chains of AGPs. Furthermore, the galactan-binding domain in CBM35 has a different ligand interaction mechanism from other sugar-binding CBM35s, including those that bind galactomannan. Specifically, we noted a Gly → Trp substitution, which affects pyranose stacking, and an Asp → Asn substitution in the binding pocket, which recognizes β-linked rather than α-linked Gal residues. These findings should facilitate further structural analysis of AGPs and may also be helpful in engineering designer enzymes for efficient biomass utilization.

Arabinogalactan proteins (AGPs) are proteoglycans characteristically localized in the plasma membrane, cell wall, and intercellular layer of higher land plants (1), in which they play functional roles in growth and development (2). The carbohydrate moiety of AGPs is composed of a β-1,3-d-galactan main chain and β-1,6-d-galactan side chain, decorated with arabinose, fucose, and glucuronic acid residues (1, 2). The chain lengths and frequencies of side chains are different among plant species, organs, and stages of development (3), and the overall structures of the carbohydrate moieties of AGPs are not yet fully understood. Degradation of polysaccharides using specific enzymes is one approach to investigate their structures and roles. In this context, exo-β-1,3-galactanase (EC 3.2.1.145) specifically cleaves the nonreducing end β-1,3–linked galactosyl linkage of β-1,3-galactans to release d-galactose (Gal). In particular, it releases β-1,6-galactooligosaccharides together with Gal from AGPs (4, 5) and is therefore useful for structural analysis of AGPs.

The basidiomycete *Phanerochaete chrysosporium* produces an exo-β-1,3-galactanase (*Pc*1,3Gal43A; GenBank^TM^ accession no. BAD98241) that degrades the carbohydrates of AGPs when grown with β-1,3-galactan as a carbon source (6). *Pc*1,3Gal43A consists of a glycoside hydrolase (GH) family 43 subfamily 24 (GH43_sub24) catalytic domain and a carbohydrate-binding module (CBM) belonging to family 35 (designated as *Pc*CBM6 in (6)) based on the amino acid sequences in the Carbohydrate-Active enZymes (CAZy) database (RRID:SCR012935) (6, 7, 8). The properties of the enzyme have been analyzed using recombinant *Pc*1,3Gal43A expressed in the methylotrophic yeast *Pichia pastoris* (6). The CBM35 of *Pc*1,3Gal43A was characterized as the first β-1,3-galactan–binding module, and *Pc*1,3Gal43A showed typical GH43_sub24 activity. The enzyme cleaves only β-1,3 linkages of oligosaccharides and polysaccharides but produces β-1,6-galactooligosaccharides together with Gal. Thus, *Pc*1,3Gal43A specifically recognizes β-1,3–linked Gal but can accommodate β-1,6–bound side chains (6).

Glycoside hydrolases are classified into families based on sequence similarity, whereas they are also divided into two major groups according to their catalytic mechanisms (*i.e.* inverting enzymes and retaining enzymes) (9, 10). Inverting enzymes typically utilize two acidic residues that act as an acid and a base, respectively, and a hydroxyl group connected to anomeric carbon inverts from the glycosidic linkage after the reaction. GH43 enzymes are members of the inverting group and share conserved Glu and Asp as the catalytic acid and base, respectively (8), but GH43_sub24 enzymes lack the catalytic base Asp (8, 11, 12). In *Ct*1,3Gal43A (from *Clostridium thermocellum*), Glu^112^ was thought to be the catalytic base (13), but in BT3683 (from *Bacteroides thetaiotamicron*), Glu^367^ (corresponding to Glu^112^ of *Ct*1,3Gal43A) was found not to act as a base but to be involved in recognition of the C-4 hydroxyl group of the nonreducing terminal Gal, and instead, Gln^577^ is predicted to be the catalytic base in the form of an unusual tautomerized imidic acid (12). An example of GH lacking a catalytic base, endoglucanase V from *P. chrysosporium* (*Pc*Cel45A), is already known, and based on the mechanism proposed for this enzyme, it is possible that tautomerized Gln functions as a base in GH43_sub24 or that this Gln stabilizes nucleophilic water. *Pc*Cel45A lacks the catalytic base Asp that is conserved in other GH45 subfamilies (14), but it uses the tautomerized imidic acid of Asn as the base, as indicated by neutron crystallography (15). However, it is difficult to understand the situation in GH43_sub24, because no holo structure with a ligand at the catalytic center has yet been solved in this family. Moreover, no structure of eukaryotic GH43_sub24 has yet been reported.

The CBM35 module is composed of ∼140 amino acids. This family includes modules with various binding characteristics and decorated with xylans, mannans, β-1,3-galactans, and glucans (16, 17, 18, 19, 20, 21). The family members are divided into four clusters based on their sequences and binding specificities (17). The structures of CBM35s binding with xylan, mannan, and glucan have already been solved (16, 17, 18, 19, 20, 21), but no structure of β-1,3-galactan–binding CBM35 has yet been reported.

In the present work, we solved the apo and liganded structures of *Pc*1,3Gal43A. Based on the results, we discuss the catalytic mechanism and the mode of ligand binding to CBM35 in the two-domain structure.

## Results

### Overall structure of Pc1,3Gal43A

The crystal structure of the SeMet derivative of *Pc*1,3Gal43A was first determined by means of the multiwavelength anomalous dispersion method, and this was followed by structure determination of the ligand-free WT, the WT bound with Gal (WT_Gal), the E208Q mutant co-crystallized with β-1,3-galactotriose (Gal3; E208Q_Gal3), and the E208A mutant co-crystallized with Gal3 (E208A_Gal3). Data collection statistics and structural refinement statistics are summarized in Table 1, Table 2, respectively.

**Table 1:** Data collection statistics Values in parentheses are for the highest-resolution shell.

Data	WT	SeMet				WT Gal3 soaking	E208Q Gal3 co-crystal	E208A Gal3 co-crystal
		Peak	Edge	Low remote	High remote			
Space group	*P*1	*P*2_1_	*P*2_1_	*P*2_1_	*P*2_1_	*P*2_1_2_1_2_1_	*P*2_1_	*P*3_2_21
**Unit-cell parameters**								
*a*, *b*, *c* (Å)	40.5, 66.3, 74.0	66.4, 50.5, 75.8				50.8, 66.6, 106.4	66.1, 50.4, 75.7	156.7, 156.7, 147.7
α, β, γ (degrees)	72.0, 84.7, 82.1	90.0, 111.9, 90.0				90.0, 90.0, 90.0	90.0, 111.3, 90.0	90.0, 120.0, 90.0
Beam line	PF BL-5	PF BL-6A	PF BL-6A	PF BL-6A	PF BL-6A	PF-AR NW12	PF-AR NE3	PF-AR NE3
Detector	ADSC Q315	ADSC Q4R				ADSC Q210	ADSC Q270	ADSC Q270
Wavelength (Å)	0.90646	0.97882	0.97950	0.98300	0.96400	1.0000	1.0000	1.0000
Resolution (Å)	50–1.40(1.45–1.40)	50.0–1.80 (1.86–1.80)	50.0–2.00 (2.07–2.00)	50.0–2.00 (2.07–2.00)	50.0–2.00 (2.07–2.00)	100.0–1.50 (1.55–1.50)	50.0–2.50 (2.54–2.50)	100.0–2.30 (2.38–2.30)
*R*_sym_	0.054 (0.370)	0.079 (0.672)	0.061 (0.307)	0.060 (0.303)	0.062 (0.307)	0.046 (0.109)	0.143 (0.399)	0.167 (0.627)
Completeness (%)	95.6 (89.0)	100.0 (99.9)	100.0 (100.0)	100.0 (100.0)	100.0 (100.0)	97.5 (94.9)	96.2 (83.0)	99.1 (92.0)
Multiplicity	3.8 (3.1)	14.0 (12.6)	7.2 (6.9)	7.2 (6.9)	7.2 (7.0)	9.2 (8.9)	4.4 (3.0)	9.7 (5.1)
Average *I*/σ(*I*)	24.4 (2.8)	36.6 (4.7)	30.9 (8.3)	30.8 (8.2)	31.3 (8.2)	48.9 (21.0)	13.5 (2.7)	17.9 (2.7)
Unique reflections	136,692 (12,747)	43,643 (4,353)	31,744 (3,139)	31,760 (3,144)	31,780 (3,146)	57,278 (5,493)	16,007 (702)	92,497 (8,510)
Observed reflections	520,085	613,162	227,158	228,381	228,595	524,957	69,939	900,469
*Z*	2	1				1	1	4

**Table 2:** Refinement statistics Values in parentheses are for the highest-resolution shell.

Data	WT	WT_Gal	E208Q_Gal3	E208A_Gal3
Resolution range	7.997–1.398 (1.448–1.398)	41.56–1.500 (1.554–1.500)	29.79–2.499 (2.588–2.499)	30.66–2.300 (2.382–2.300)
Completeness (%)	95.46 (87.82)	97.51 (94.80)	96.41 (85.67)	98.78 (92.17)
Wilson *B*-factor	12.76	10.11	29.91	30.40
Reflections used in refinement	136,655 (12,497)	57,105 (5,474)	15,762 (1,381)	92,011 (8,507)
Reflections used for *R*-free	6,862 (630)	2,884 (272)	799 (64)	4,568 (441)
*R*-work (%)	15.47 (22.50)	13.43 (12.71)	16.62 (25.54)	16.10 (22.39)
*R*-free (%)	18.56 (26.28)	16.00 (17.93)	24.39 (42.53)	21.43 (28.28)
**No. of nonhydrogen atoms**	7,966	3,923	3,576	14,570
Macromolecules	6,615	3,290	3,235	12,886
Ligands	109	121	114	678
Solvent	1,242	512	227	1,006
Protein residues	2,106	427	428	1,708
r.m.s. (bonds)	0.008	0.006	0.008	0.011
r.m.s. (angles)	1.22	0.87	0.94	1.05
Ramachandran favored (%)	97.29	97.41	94.13	95.76
Ramachandran allowed (%)	2.71	2.59	5.87	4.24
Ramachandran outliers (%)	0	0	0	0
Rotamer outliers (%)	0.81	0.55	0.29	0.36
Clash score	2.06	1.95	6.94	3.50
**Average *B*-factor (Å^2^)**	17.21	12.45	30.48	32.98
Macromolecules	14.97	10.57	29.77	31.60
Ligands	29.38	23.33	52.26	56.11
Solvent	28.09	22.02	29.74	35.03
**PDB code**	7BYS	7BYT	7BYV	7BYX

The recombinant *Pc*1,3Gal43A molecule is composed of a single polypeptide chain of 428 amino acids (Gln^21^–Tyr^448^) with two extra amino acids, Glu^19^ and Phe^20^, derived from the restriction enzyme cleavage site, which are disordered and thus were not observed. The protein is decorated with *N*-glycans because it was expressed in *Pichia* yeast. Up to three sugar chains are attached at Asn^79^, Asn^194^, and Asn^389^; the attached chains vary in position and structure, and most contain one or two GlcNAc moieties.

**Figure 1:**
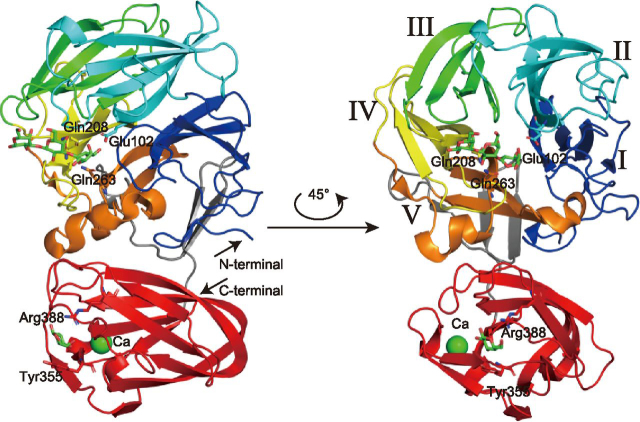
**Overall structure of *Pc*1,3Gal43A.** In the three-dimensional structure of *Pc*1,3Gal43A, the five blades of the catalytic domain are shown in *blue* (Gln^21^–Leu^87^), *cyan* (Ser^88^–Asp^155^), *green* (Ser^156^–Gly^204^), *yellow* (Ala^205^–Ser^247^), and *orange* (Ala^248^–Asp^297^) with successive *roman numerals*. The CBM (The^326^–Val^448^) is shown in *orange*. The linker connecting the two domains (Phe^298^–Gly^325^) is shown in *gray*.

*Pc*1,3Gal43A is composed of two domains, and ligands introduced by soaking or co-crystallization are located in a subsite of the catalytic domain or the binding site of CBM35 (Fig. 1). The N-terminal catalytic domain consists of a five-bladed β-propeller (Gln^21^–Gly^325^), as in other GH clan-F enzymes, and the C-terminal domain (*Pc*CBM35) takes a β-jellyroll fold (Thr^326^–Tyr^448^) structure, as in previously reported CBM35s (16, 17, 18, 19, 20, 21, 22, 23, 24, 25). *Pc*CBM35 contains one calcium ion near the end of the first β-strand on a different domain surface from the plane to which the ligand binds (Fig. 1). The structure of *Pc*CBM35 is similar to those of other known CBM35s. The interface area is 686 Å^2^ and includes many water molecules. The PDBePISA server (RRID:SCR015749) indicates that the enzyme forms a complex in the crystal, but this is an effect of crystallization, and the enzyme exists as a monomer in solution (data not shown).

### Sugar-binding structure of the Pc1,3Gal43A catalytic domain

The five-bladed β-propeller exhibits an almost spherical structure, and two central cavities are located at the ends of the pseudo-5-fold axis (Fig. 1). One of them contains the catalytic site and it is common in almost all GH43 enzymes. The catalytic site is located in the center of the five-bladed β-propeller, whose blades are formed by Gln^21^ or Asn^22^–Leu^87^ (*I* in Fig. 1), Ser^88^–Asp^155^ (*II* in Fig. 1), Ser^156^–Gly^204^ (*III* in Fig. 1), Ala^205^–Ser^247^ (*IV* in Fig. 1), and Ala^248^–Asp^297^ (*V* in Fig. 1).

**Figure 2:**
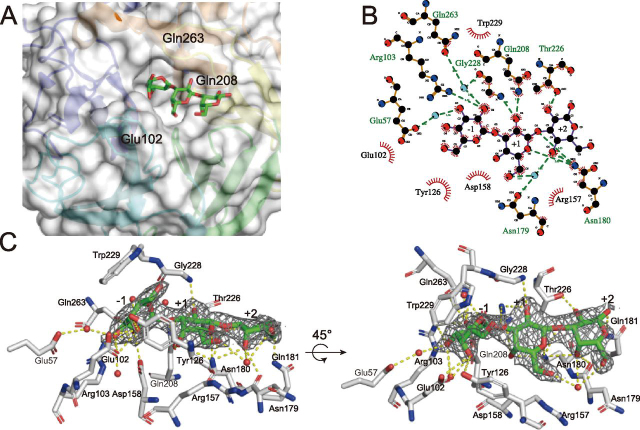
**Gal3-binding mode at the catalytic site.***A*, *surface structure* of the catalytic center. Gal3 is represented as *green* (carbon) and *red* (oxygen) sticks. *B*, schematic diagram showing the interaction mode at the catalytic center. *Black*, *red*, and *blue*, carbon, oxygen, and nitrogen, respectively. *Red lines* indicate the hydrophobically interacting residues. This diagram was drawn with LigPlot+ (version 1.4.5). *C*, the 2*F_o_* − *F_c_* omit map is drawn as a *blue mesh* (0.8σ). Residues are shown in *white* (carbon), *red* (oxygen), and *blue* (nitrogen). Gal3 is shown in *green* (carbon) and *red* (oxygen). *Yellow dots*, hydrogen bonds and/or hydrophobic interaction; *red spheres*, water molecules interacting with ligands or residues.

As shown in Fig. 2, the Gal3 molecule co-crystallized with the E208Q mutant occupies subsites −1, +1, and +2 of the catalytic site, from the nonreducing end to the reducing end. Gal_−1_ is located at the bottom of the catalytic cavity, and Gal_+1_ and Gal_+2_ extend linearly outwards. Gal_+1_ is half-buried in the cavity, whereas Gal_+2_ is exposed at the surface (Fig. 2*A*).

Gal_−1_ adopts a ^1^*S*_3_ skew boat conformation and interacts with many residues via hydrogen bonds and hydrophobic interactions. As shown in Fig. 2 (*B* and *C*), the C-2 hydroxyl group of Gal_−1_ forms hydrogen bonds with NH_2_ of Arg^103^ and with OE1 of Gln^263^ via water. In addition, this water molecule is bound with O of Gly^228^. The C-3 hydroxyl group of Gal_−1_ also forms a hydrogen bond with OE2 of Glu^57^ via water. Glu^102^, Tyr^126^, Asp^158^, Gln^208^, Thr^226^, Trp^229^, and Gln^263^ interact with Gal3 through hydrophobic interactions. Notably, Trp^229^ supports the flat C3-C4-C5-C6 structure of Gal_−1_, and Tyr^126^ recognizes the C-6 methylol and C-4 hydroxyl groups, whereas Glu^102^ recognizes the C-3 hydroxyl and C-4 hydroxyl groups. In Gal_+1_ (as shown in Fig. 2, *B* and *C*), the C-2 hydroxyl group forms a hydrogen bond with NE2 of Gln^208^ and N of Gly^228^, whereas O5 forms a hydrogen bond with ND2 of Asn^180^, and C-6 hydroxyl group forms a hydrogen bond with OD1 of Asn^179^ via water. Tyr^126^, Arg^157^, Asn^180^, and Gln^208^ interact hydrophobically with Gal. In Gal_+2_ (Fig. 2, *B* and *C*), the C-2 and C-4 hydroxyl groups form hydrogen bonds with OG1 of Thr^226^ and ND2 of Asn^180^, respectively. In addition, Thr^226^ interacts with Gal_+2_ through hydrophobic interaction. Furthermore, the glycosidic oxygen between Gal_+1_ and Gal_+2_ interacts with ND2 of Asn^180^ through a hydrogen bond.

In the structure of WT_Gal, one Gal was found at subsite −1, taking a ^4^*C*_1_ chair conformation with α-anomeric conformation of the C-1 hydroxyl group (data not shown). The binding mode of Gal_−1_ is almost the same as that in E208Q_Gal3, but the C-1 hydroxyl group in the axial position forms hydrogen bonds with Gly^228^ and Gln^263^. No Gal3 molecule was observed at the catalytic domain in the structure of the Gal3 co-crystallized E208A mutant.

To identify the catalytic residues, we examined the relative activity of WT and the six mutants toward β-1,3-galactobiose (Gal2) and Gal3. WT showed 5.58 ± 0.35 and 11.15 ± 0.39 units of activity (μmol of Gal/min/nmol of enzyme) toward Gal2 and Gal3, respectively, whereas the six mutants showed no detectable activity (Fig. S1), suggesting that these residues are all essential for the catalysis.

### Sugar-binding structure of CBM35 in Pc1,3Gal43A

*Pc*1,3Gal43A has one CBM35 domain at the C terminus. We previously reported that this enzyme has a CBM6-like domain (6), but it has been reclassified into the CBM35 family (7). The β-jellyroll fold domain is accompanied by a single calcium ion–binding site on a domain surface different from the surface to which the ligand at the end of the first β chain binds, and this corresponds to a conserved calcium ion–binding site in CBM35s. Some CBM35 modules bind another calcium ion at a site at the top of domain (16), but *Pc*CBM35 lacks this second calcium ion–binding site (Fig. 1).

**Figure 3:**
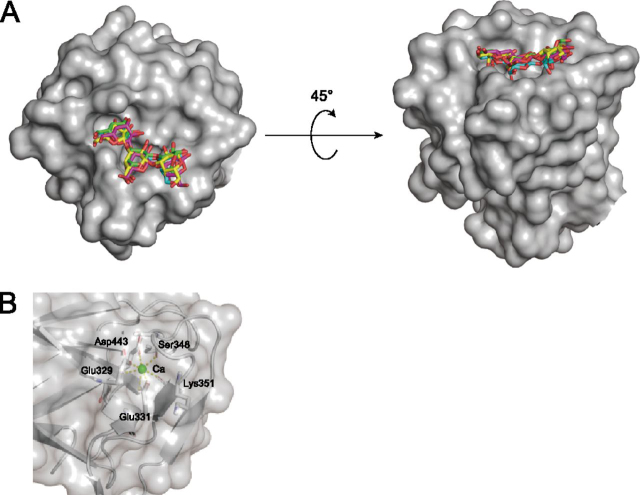
**Surface structures of the CBM.***A*, substrate-binding mode at CBM35. *Green*, *cyan*, *magenta*, and *yellow*, carbons of chains A, B, C, and D of E208A_Gal3, respectively; *red*, oxygen. *Left*, nonreducing end of Gal3; *right*, reducing end. *B*, calcium ion–binding mode at CBM35. Calcium ion is represented as *green spheres*, and interacting residues are shown as *stick models*. *Yellow dots*, interaction.

**Figure 4:**
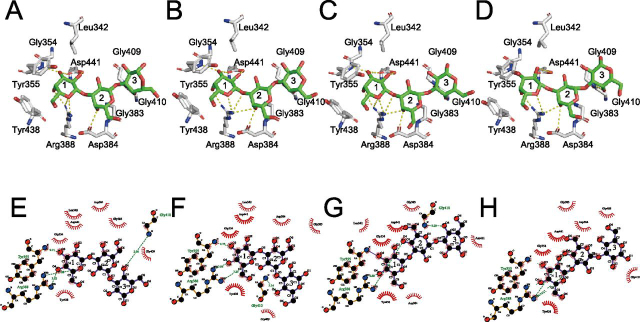
**Ligand interaction mode at CBM35.***A* and *E*, *B* and *F*, *C* and *G*, and *D* and *H*, chains A, B, C, and D of E208A, respectively. *A–D*, interaction modes between ligand and CBM35 residues. Atoms are indicated in the *same colors* as in Fig. 2. *E–G*, schematic diagram showing the interaction mode at CBM35. Atoms are indicated in the *same colors* as in Fig. 2. Sugar-binding sites are named Gal_site 1, Gal_site 2, and Gal_site 3 from the nonreducing end of the sugar, and in this figure, they are labeled *1*, *2*, and *3*, respectively.

In E208A_Gal3, electron density of Gal3 was observed in the ligand-binding site of *Pc*CBM35. As illustrated in Fig. 3*A* and Fig. S2, 2*F_o_* − *F_c_* omit maps showed that the binding mode of *Pc*CBM35 with ligands is “exo-type,” corresponding to type-C CBM (26). The asymmetric unit of E208A_Gal3 contained four *Pc*1,3Gal43A molecules, and each molecule binds to the nonreducing end of Gal3 (called Gal_site 1), as in other CBM35 modules. However, the middle Gal (Gal_site 2) and the reducing end Gal (Gal_site 3) are found in two main locations (Fig. 3), although residues involved in the interactions with the ligand in each molecule were mostly shared. The Gal_site 1 forms hydrogen bonds with Tyr^355^ and Arg^388^ and interacts hydrophobically with Leu^342^, Gly^354^, Tyr^438^, and Asp^441^. The Gal_site 2 interacts hydrophobically with Gly^383^ and Asp^384^. The main ligand interaction in the Gal_site 3 involves Gly^409^ and Gly^410^, but in addition to these residues, Asn^411^ is also involved in ligand recognition in chain C (Fig. 4).

### Ensemble refinement

**Figure 5:**
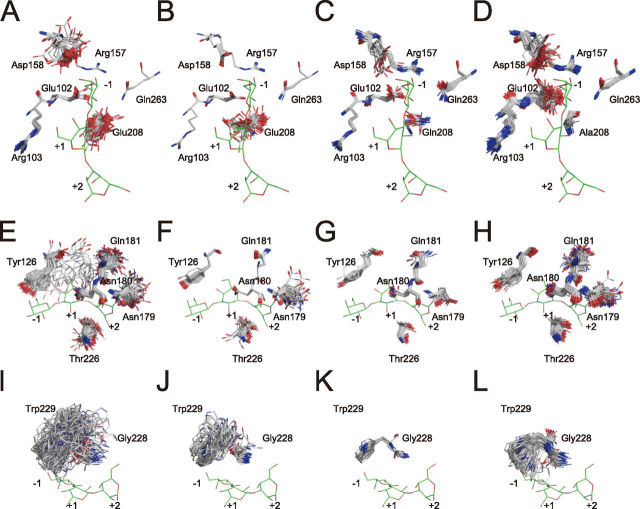
**Results of ensemble refinement at the catalytic site.** Each model is divided into three parts for clarity. *A* (*E* and *I*), *B* (*F* and *J*), *C* (*G* and *K*), and *D* (*H* and *L*) show WT, WT_Gal, E208Q_Gal3, and E208A_Gal3, respectively. Although WT and E208A_Gal3 contained multiple molecules in an asymmetric unit, the results obtained with multiple molecules were considered as an ensemble of one molecule in the present study. *Letters* indicate the chain names. Atoms are indicated in the *same colors* as in Fig. 2. Gal3 of the structure of E208Q_Gal3 obtained by X-ray crystallography is arranged in each figure to maximize ease of comparison.

**Figure 6:**
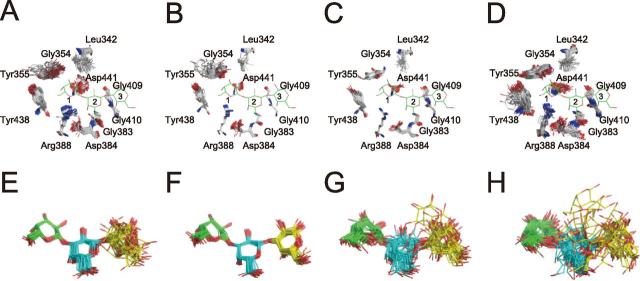
**Results of ensemble refinement at the CBM ligand-binding site.***A*, *B*, *C*, and *D*, residues related to ligand interaction. In this figure, Gal3 of chain A of refined E208A_Gal3 is drawn for comparison. *E*, *F*, *G*, and *H*, the ligands of each chain. *Green*, *cyan*, and *yellow* are used in order from the nonreducing terminal Gal. *A* and *E*, *B* and *F*, *C* and *G*, and *D* and *H* represent chains A, B, C, and D of E208A_Gal3, respectively. Atoms are indicated in the *same colors* as in Fig. 2.

To understand the fluctuation of ligands, ensemble refinements were performed with the refined models. This method produces ensemble models by employing a combination of X-ray structure refinement and molecular dynamics. These models can simultaneously account for anisotropic and anharmonic distributions (27). Four different pTLS values of 0.6, 0.8, 0.9, and 1.0% were set for each model. Table 3 shows the statistical scores of the refinement with the most appropriate pTLS value for each model. Focused views of the catalytic site in the catalytic domain and the ligand-binding site of the CBM are shown in Figure 5, Figure 6, respectively. Note that structures containing multiple molecules in the asymmetric unit (WT and E208A_Gal3) are found for all molecules in this paper.

**Table 3:** Refinement statistics of ensemble refinement Values in parentheses are for the highest-resolution shell.

Data	WT	WT_Gal	E208Q_Gal3	E208A_Gal3
Resolution range	7.997–1.398 (1.448-1.398)	41.56–1.500 (1.554–1.500)	29.79–2.499 (2.588–2.499)	30.66–2.300 (2.382– 2.300)
Completeness (%)	95.97 (82)	97.52 (95)	96.47 (88)	98.93 (87)
pTLS (%)	0.9	0.9	0.9	1.0
Tx	1.0	0.9	0.3	0.4
Wilson *B*-factor	12.8	10.1	29.9	30.4
Reflections used in refinement	136,649	57,112	15,759	91,994
Reflections used for *R*-free	6,862	2,885	799	4,569
*R*-work	13.81 (24.36)	12.08 (10.68)	17.82 (24.73)	15.92 (22.56)
*R*-free	17.08 (26.30)	15.29 (17.10)	23.33 (32.75)	20.71 (28.84)
r.m.s. (bonds)	0.008	0.010	0.007	0.008
r.m.s. (angles)	1.171	1.312	1.078	1.090
Ramachandran favored (%)	94.06	95.39	88.98	92.62
Ramachandran allowed (%)	5.08	4.03	9.19	7.24
Ramachandran outliers (%)	0.86	0.58	1.83	0.74
Rotamer outliers (%)	7.45	7.00	11.05	7.85
Clash score	0	0	0	0
**Average *B*-factor (Å^2^)**	13.65	9.55	28.32	32.83
Macromolecules	13.63	9.54	28.30	32.66
Ligands	14.97	9.82	28.98	36.05
Molprobity score	1.56	1.45	1.87	1.64
Model number	100	103	20	34

In the catalytic site, the vibration levels of some residues were significantly different between the apo and holo forms. As shown in Fig. 5, Tyr^126^, Arg^157^, Asp^158^, Asn^179^, Asn^180^, Gln^181^, Trp^229^, and Gln^263^ in the liganded structures (Fig. 5, *B*, *C*, *F*, *G*, *J*, and *K*) showed smaller vibrations than in the apo structures (Fig. 5, *A*, *D*, *E*, *H*, *I*, and *L*). These results indicate that side-chain fluctuations converge upon ligand binding. Comparison of the Gal-bond structure (*i.e.* WT_Gal; Fig. 5, *B*, *F*, and *J*) with the Gal3-bond structure (*i.e.* E208Q_Gal3; Fig. 5, *C*, *G*, and *K*) showed that the fluctuations of Glu(Gln)^208^, Asn^179^, and Thr^226^ of E208Q_Gal3 were smaller than these in WT_Gal. Therefore, it can be inferred that these residues recognize the ligands at the plus subsites. The catalytic acid, Glu^208^, has two major conformations in WT and WT_Gal. These two conformations were also reported in the BT3683 structure (12). Thus, the movement of this residue appears to be important for catalysis. Gln^263^ shows one conformation (Fig. 5, *A–D*) that is identical to the result of the ensemble refinement of Asn^92^, known as imidic acid in *Pc*Cel45A (Fig. S3). Glu^102^ may distinguish nonreducing terminal Gal, because it interacts with the axial C-4 hydroxyl group of Gal_−1_ (12). The vibration degree of Glu^102^ was different between WTs and mutants, so its conformation does not depend on the ligand localization, but reflects interaction with Glu^208^, which serves as a general acid. Asp^158^ of WT and E208A_Gal3 show greater vibration than WT_Gal and E208Q_Gal3. The role of Asp^158^ is thought to be a p*K_a_* modulator; therefore, its function and conformational stability might be related. Focusing on Fig. 5 (*I–L*), there were large differences in the fluctuation level of Trp^229^. E208Q_Gal3 (Fig. 5*K*) showed small movements of Trp^229^, but other structures showed much larger fluctuations (Fig. 5, *I*, *J*, and *L*). These results suggest that this Trp is normally flipped and forms a π-π interaction to anchor the ligand in the proper position upon arrival. A histogram of the dihedral angle is shown in Fig. S4.

**Figure 7:**
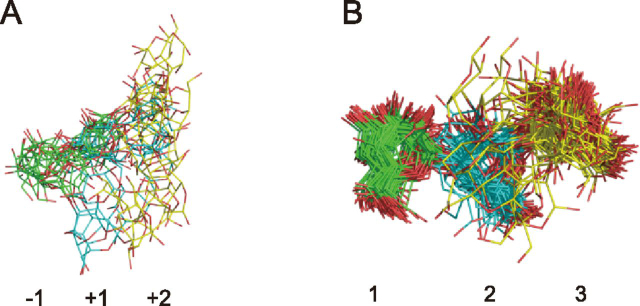
**Ligand conformation of ensemble refinement at a glance.***A*, ligand conformation of E208Q_Gal3 ensemble model. *B*, ligand conformation of E208A_Gal3 ensemble models with four chains aligned. *Green*, *cyan*, and *yellow* are used in order from the nonreducing terminal Gal.

As regards the ligand-binding site of the CBM, a comparison of each chain of the E208A_Gal3 asymmetric unit showed no significant difference in the vibration levels of each residue involved in ligand binding (Fig. 6). However, ensemble refinement revealed that Gal_site 1 and Gal_site 2 do not show huge fluctuations, whereas Gal_site 3 has many conformations. They include the same conformation of each Gal chain in X-ray crystallography. Interestingly, a spatial difference in fluctuations was observed between ligands bound to the catalytic site and to the ligand-binding site of CBM35 (Fig. 7). At the catalytic site, Gal_−1_ is anchored in the appropriate position, and Gal_+2_ appears to fluctuate in a planar fashion as it interacts with the surrounding residues. In the CBM, it was inferred that Gal_site 1 is fixed and Gal_site 3 is adsorbed at the appropriate location at the binding site while fluctuating in three dimensions.

## Discussion

Most exo-β-1,3-galactanases belonging to GH43_sub24 possess CBMs that can be classified into CBM35 or CBM13 (8). In this study, we elucidated the structure of a β-1,3-galactan–binding module for the first time by solving the structure of a GH43_sub24 containing CBM35 and obtained the ligand-bound structures of both the catalytic and sugar-binding domains of *Pc*1,3Gal43A. This is also the first study to reveal the structure of a eukaryotic exo-β-1,3-galactanase. This information will be useful to understand how the CBM35 module interacts with β-1,3-galactan in combination with the GH43_sub24 catalytic module.

### How does Pc1,3Gal43A hydrolyze β-1,3-galactan?

Although catalytic residues such as Glu and Asp are conserved in GH43 as a catalytic acid and base, respectively, GH43_sub24 lacks such a base residue. Cartmell *et al.* (12) suggested that GH43_sub24 may use Gln in the base role via conversion to imidic acid or use an exogenous base or utilize the Grotthuss mechanism of catalysis (8, 12). In this study, we measured the enzyme activity of six variants of the three residues speculated to be involved in the catalytic reaction. As shown in Fig. S1, production of Gal by the mutants was not detected by means of HPLC analysis, suggesting that all three residues are essential for the catalytic activity of *Pc*1,3Gal43A. Glu^102^, Glu^208^, and Gln^263^ are speculated to serve in C-4 hydroxyl group recognition, as a catalytic acid, and as a catalytic base, respectively. These residues are well-conserved in GH43_sub24, as shown in Fig. S5.

**Figure 8:**
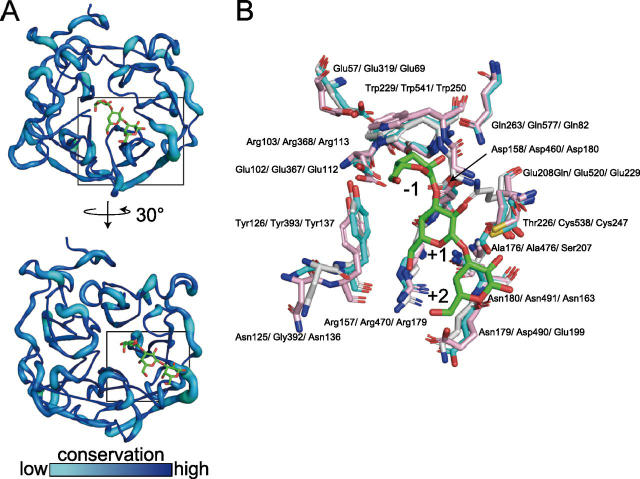
**Catalytic domain structure comparison.***A*, visualization of the degree of preservation of GH43_sub24. The degree of conservation of amino acid residues in the catalytic domain of GH43_sub24 was visualized using the ConSurf server (RRID:SCR002320), the query for BLAST was set to *Pc*1,3Gal43A, and the conservation degree was analyzed based on 150 amino acid sequences in the ConSurf server (47,48, 49, 50,51). The conservation degree is shown in *graded color*. Preservation degrees are shown in a gradient with *cyan* for the lowest degree of preservation and *blue* for the highest. *B*, catalytic domain comparison of *Pc*1,3Gal43A and two GH43_sub24 galactanases. Shown are the catalytic centers of E208Q_Gal3 of *Pc*1,3Gal43A (*white*, PDB code 7BYV), BT3683 (*cyan*, PDB code 6EUI), and *Ct*1,3Gal43A (*pink*, PDB code 3VSF). *Red*, *blue*, and *yellow*, oxygen atoms, nitrogen atoms, and sulfur atoms, respectively. Residue names are shown for *Pc*1,3Gal43A/BT3683/*Ct*1,3Gal43A.

In GH43_sub24, only bacterial enzyme structures have been solved so far (http://www.cazy.org/GH43_24.html). To understand the catalytic mechanism of *Pc*1,3Gal43A, we compared its structure with those of BT3683 and *Ct*1,3Gal43A (Fig. 8). Most of the residues that interact with ligands are conserved in these three enzymes. In subsite −1, all residues, Glu^57^, Glu^102^, Arg^103^, Tyr^126^, Asp^158^, Glu^208^, Trp^229^, and Gln^263^, of *Pc*1,3Gal43A are conserved, indicating that the binding mode at subsite −1 is fully conserved in GH43_sub24. Based on the results of ensemble refinement, Trp^229^ showed huge fluctuation, especially in the apo structure (Fig. 5, *I–L*). Trp^541^ of BT3683, which corresponds to Trp^229^ of *Pc*1,3Gal43A, has a polar interaction with Gal (12). Trp^229^ fluctuates in solution and plays a role in holding the substrate at the catalytic site through polar interactions. On the other hand, Asn^179^ and Thr^226^ of *Pc*1,3Gal43A are replaced by Asp^490^ and Cys^538^ in BT3683 and by Glu^199^ and Cys^247^ in *Ct*1,3Gal43A. Because all of these enzymes can accommodate a β-1,6–branched side chain (6, 12, 28), we considered that these residues are not related to the mechanism of side-chain accommodation.

**Figure 9:**
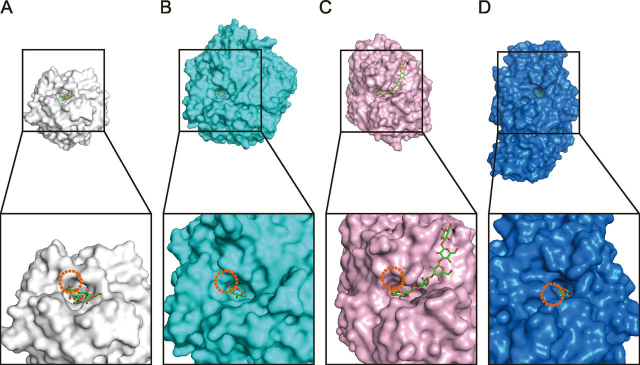
**Structure comparison of the catalytic sites of *Pc*1,3Gal43A (*A*), GH55 exo-**β**-1,3-glucanase from *P. chrysosporium* (*B*; *Pc*Lam55A; PDB code**3EQO), GH55 exo-β**-1,3-glucanase from *Streptomyce*s sp. SirexAA-E (C; SacteLam55A; PDB code**4PF0), **and GH3 β-glucosidase from *H. jecorina* (*D*, *Hj*Cel3A; PDB code**3ZYZ). *A*, *B*, and *C* hydrolyze the main chain of β-1,3-galactan or β-1,3-glucan, bypassing β-1,6–branched side chains (6, 29, 30). *D* hydrolyzes four types of β-bonds, and it does not bypass side chains (31, 52). The *top panel* shows the overall surface structure, and the *bottom panel* shows an *enlarged view* of the catalytic region. *Orange dashed circles*, space near the C-6 position of Gal or glucose at the nonreducing end.

The bypass mechanism of *Pc*1,3Gal43A, which enables accommodation of the β-1,6-galactan side chain so that the β-1,3-galactan main chain can be cleaved, appears to depend on the orientation of the C-6 methylol group of Gal3 at each subsite. The C-6 methylol group of Gal_−1_ is exposed to the solvent, so that the side chain can be accommodated externally. The C-6 methylol groups of Gal_+1_ and Gal_+2_ are also exposed to the solvent, so that the enzyme should be able to cleave the β-1,3 linkage of continuously β-1,6–substituted galactan, and a similar situation has been reported for BT3683 (12). Moreover, there are spaces near the nonreducing terminal Gal in these enzymes (12). This enables the enzymes to degrade the main chain, even if the side chain contains multiple carbohydrates. Similarly, β-1,3-glucanases belonging to GH55 also bypass the β-1,6-glucan side chain and degrade β-1,3-glucan from the nonreducing end (29, 30). Comparing the surface structure of the catalytic site of *Pc*1,3Gal43A with that of these GH55 exo-β-1,3-glucanases from *P. chrysosporium* (*Pc*Lam55A), we see that *Pc*1,3Gal43A has a small pocket-like space capable of accepting the C-6 side chain of Gal at subsite −1 (Fig. 9, *A* and *B*). In addition, the C-6 methylol group of Gals, located at the positive subsites of *Pc*1,3Gal43A, are exposed to solvent in a similar manner to that reported for SacteLam55A, GH55 exo-β-1,3-glucanase from *Streptomyce*s sp. SirexAA-E (Fig. 9, *A* and *C*). Structures capable of accepting nonreducing terminal Gal with β-1,6–linked Gal are conserved among GH43_sub24 of known structure (Fig. 8 and Fig. S5). In the nonbypassing GH3 *Hypocrea jecorina* β-glucosidase (*Hj*Cel3A), the C-6 hydroxyl group of nonreducing glucose is oriented toward the enzyme, introducing steric hindrance (Fig. 9*D*) (31). In other words, enzymes bypassing side chains have a space adjacent to C-6 of the nonreducing terminal sugar, and the positive subsites are particularly wide, allowing side chains of the substrate to be accommodated. In contrast, enzymes unable to bypass the side chain have no space next to the −1 subsite and have a narrow entrance to the catalytic site, so that they are unable to accommodate side chains (Fig. 9*D*).

Although the electron density of Gal3 was observed in the present study, *Pc*1,3Gal43A is proposed to have four subsites ranging from −1 to +3, based on biochemical experiments (6). As mentioned above, although *Pc*1,3Gal43A has a structure capable of accepting the C-6 side chain, its degradation activity toward β-1,3/1,6-galactan is only approximately one-fifth that of the linear β-1,3-galactan (6). This difference in reactivity may be due to the structure of the sugar. The β-1,3-galactan in solution has a right-handed triple helical structure with 6–8 Gal residues per turn (32, 33), with the C-6 methylol group pointing outward to avoid collisions between the β-1,6–bonded Gal side chains (32). However, as shown in Fig. S6, Gal3 bound to the catalytic site of *Pc*1,3Gal43A is anchored to the enzyme, so that the helix of the glycans differs from the usual state in solution. Therefore, the reason why the hydrolytic activity of *Pc*1,3Gal43A toward β-1,3/1,6-galactan is lower than that toward β-1,3-galactan may be interference between the β-1,6-Gal side chains as a result of changes in the helical state of the main chain.

### How does PcCBM35 recognize β-1,3-galactan?

**Figure 10:**
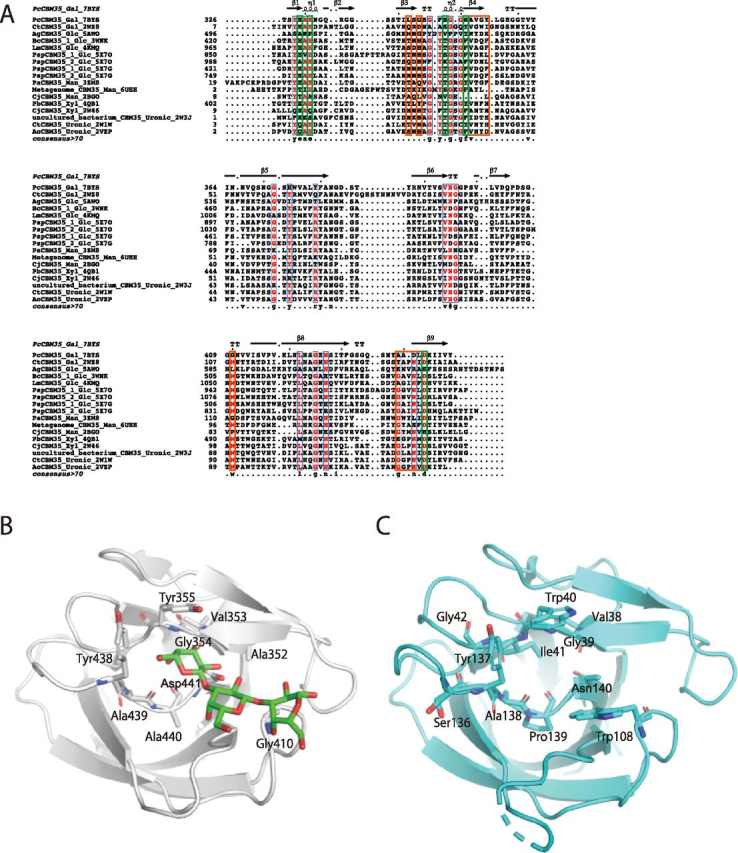
**Sequence alignment of known CBM35s (*A*) and structure comparison between CBM35s of *Pc*1,3Gal43A (*B*) and Cte_2137 (*C*).***A*, taxon names are shown as scientific names, ligand specificity, and PDB code only for brevity. When the same enzyme contains two CBM35 domains, the taxon name is indicated with *1* on the N terminus and *2* on the C terminus. *Gal*, *Glc*, *Man*, *Xyl*, and *Uronic*, ligand specificities for Gal, glucose, mannose, xylose, and glucronic acid and/or galacturonic acid, respectively. Among these, 3ZM8, 6UEH, and 2BGO, which bind to Man, are type B CBMs, which show endo-type binding, whereas the other 14 are all type C CBMs, which show exo-type binding. The alignment was built by using MUSCLE on MEGAX: Molecular Evolutionary Genetics Analysis (53, 54), and the figure was generated with ESPrint 3.0 (RRID:SCR006587) (55). *Orange* and *green boxes* represent ligand-binding and calcium ion–binding residues, respectively. *B* and *C*, ligand-binding residues of *Pc*1,3Gal43A (chain A of E208A_Gal3) and Cte_2137 (PDB code 2WZ8). *Red* and *blue*, oxygen and nitrogen, respectively. The *green stick model* represents Gal3.

Although the amino acid sequence similarity of CBM35s is not so high, important residues involved in ligand binding are well-conserved (17). The modules belonging to CBM35 can be divided into four clades according to the mode of ligand binding, and the diversity in ligand binding and in the calcium ion–coordinating residue account for the various ligand-binding specificities (17) (Fig. 10*A*). Moreover, the residues involved in ligand binding of *Pc*CBM35 differ from those of CBM35, which binds to α-Gal of galactomannan. This CBM is one part of a protein predicted to be the β-xylosidase of *C. thermocellum* cellulosomal protein (Cte_2137; Fig. 10), which belongs to the same cluster as *Pc*CBM35 (17). There are some differences between the residues interacting with α-Gal of Cte_2137 and those interacting with β-Gal of *Pc*CBM35. For instance, the regions of Ala^352^–Tyr^355^ and Tyr^438^–Asp^441^ of *Pc*CBM35 correspond to Val^39^–Gly^42^ and Ser^136^–Asn^140^ of Cte_2137, which are related to ligand specificity (Fig. 10). Especially, Asn^140^ of Cte_2137 is not conserved but replaced by Asp^441^ in *Pc*CBM35 and is located at the bottom of the ligand-binding site. Furthermore, Trp^108^ of Cte_2137 plays a key role in sacking the pyranose ring (17), whereas in CBM35 of *Pc*1,3Gal43A, this Trp residue is replaced with Gly (Fig. 10*B*). In other words, although *Pc*CBM35 and Cte_2137 are in the same cluster, the residues involved in ligand recognition are different, and this difference affects the discrimination between β-Gal and α-Gal and between galactan and galactomannan. It is still unclear how CBM35s acquire such variation of binding specificity within a similar binding architecture. However, a detailed understanding of the molecular mechanisms of polysaccharide recognition by CBM35 will be essential for efficient utilization of various types of biomass.

In conclusion, we have determined the crystal structure of the catalytic and binding domains of *Pc*1,3Gal43A with the aim of reaching a detailed understanding of the mechanism of substrate accommodation by side chain–bypassing galactanase. *Pc*1,3Gal43A uses Glu as the catalytic acid and Gln as the catalytic base and has a structure in which the side chain of the substrate does not interfere with the catalytic reaction, thus making it possible to degrade the β-1,3-galactan main chain of AGPs despite the presence of the β-1,6-galactan side chain. Thus, although polysaccharides have a variety of molecular decorations, it appears that the structures of the degrading enzymes enable them to recognize specific features of the substrate while accommodating the variations. The introduction of mutations in substrate recognition residues to create enzymes with altered substrate recognition properties is expected to be helpful in the structural analysis of AGP glycans and also for the preparation of useful oligosaccharides.

## Experimental procedures

### Expression of Pc1,3Gal43A and its mutants

The E208Q, E208A, E102Q, E102A, Q263E, and Q263A mutants were constructed by inverse PCR using PrimeSTAR MAX (Takara, Tokyo, Japan). For crystallization, *Pc*1,3Gal43A WT, E208Q, and E208A from *P. chrysosporium* were expressed in *P. pastoris* and purified as reported previously (7). For a reactivity assay, WT and mutants were purified by using SkillPak TOYOPEARL Phenyl-650M (c.v. = 5 ml, Tosoh, Tokyo, Japan) equilibrated with 20 mm sodium acetate buffer, pH 4.0, containing 1 m ammonium sulfate, and the enzymes were eluted with 20 mm sodium acetate buffer, pH 4.0, containing 0.7 m ammonium sulfate. SeMet-labeled *Pc*1,3Gal43A was expressed as reported previously (7).

### Preparation for β-1,3-galactooligosaccharides and crystallization of Pc1,3Gal43A

Gal2 and Gal3 were prepared as reported previously (6). The protein solution was concentrated to 4.9–6.9 mg/ml and used for the crystallization setup. The WT plate crystal used for data collection was obtained from a reservoir of 2.1 m ammonium sulfate, 0.1 m citrate buffer, pH 5.5. Other WT crystals were obtained from solutions in 16% (w/v) PEG 10000, 0.1 m ammonium sulfate, 0.1 m bis-tris, pH 5.5, and 5.0% (v/v) glycerol. SeMet crystals were obtained from 16% (w/v) PEG 10000, 95 mm ammonium sulfate, 95 mm bis-tris, pH 5.5, and 4.8% (v/v) glycerol. Two types of crystals, thin plate crystals (space group *P*2_1_) and rod crystals (*P*2_1_2_1_2_1_), appeared under the same condition. Cocrystallization of the E208Q mutant with 10 mm Gal3 in 16% (w/v) PEG 10000, 95 mm ammonium sulfate, 95 mm bis-tris, pH 5.5, and 4.8% (v/v) glycerol afforded thin plate crystals. The E208A mutant was cocrystallized with 10 mm Gal3 in 0.2 m potassium nitrate, 15% (w/v) PEG 6000, 20 mm sodium citrate, pH 4.5, and 5% glycerol to afford bipyramidal crystals.

### Data collection and structure determination

Diffraction experiments for *Pc*1,3Gal43A crystals were conducted at the beamlines of the Photon Factory (PF) or Photon Factory Advanced Ring (PF-AR), High Energy Accelerator Research Organization, Tsukuba, Japan (Table 1). Diffraction data were collected using CCD detectors (Area Detector Systems Corp., Poway, CA, USA). Crystals were cryocooled in a nitrogen gas stream to 95 K. For data collection of the WT enzyme complexed with Gal3, *Pc*1,3Gal43A crystals were soaked in a drop containing 1% (w/v) Gal3 for 10 min before the diffraction experiment. The data were integrated and scaled using the programs DENZO and SCALEPACK in the HKL2000 program suite (34).

Crystal structure was determined by means of the multiwavelength anomalous dispersion method using a SeMet-labeled crystal (7). Initial phases were calculated using the SOLVE/RESOLVE program (35) from five selenium atom positions. The resultant coordinates were subjected to the automodeling ARP/wARP program (36) in the CCP4 program suite (37), and manual model building and molecular refinement were performed using Coot (version 0.8.9, University of Oxford, Oxfordshire, UK) (38), REFMAC5 (version 7.0.063, Science and Technology Facilities Council, Swindon, UK) (39), phenix.refine (40), and phenix.ensemble_refinement (27, 41, 42) in the Phenix suite of programs (version 1.13-2998-000, Lawrence Berkeley National Laboratory, Berkeley, CA, USA) (43). The refinement statistics are summarized in Table 2.

For the analyses of WT and ligand-bound structures, structural determination was conducted by the molecular replacement method with the MOLREP program (44) in the CCP4 program suite using the SeMet or ligand-free structure as the starting model. Bound sugars, water molecules, and crystallization agents were modeled into the observed electron density difference maps. Calcium ion was modeled based on the electron density map and the coordination distances. Three *N*-glycans were observed, and the identified sugars were modeled. The stereochemistry of the models was analyzed with LigPlot + (version 1.4.5) (45, 46), and structural drawings were prepared using PyMOL (version 2.2.3, Schrödinger, LLC, New York).

### Enzymatic activity assay of Pc1,3Gal43A and its mutants

To evaluate the reactivity toward Gal2 and Gal3 of WT and each mutant, 20 nm enzyme was incubated with 0.263 or 0.266 mm galactooligosaccharides in 20 mm sodium acetate, pH 5.0, for 30 min at 30 °C, respectively. The reaction was stopped by heating at 95 °C for 5 min. The supernatant was separated with 75% (v/v) acetonitrile on a Shodex Asahipak NH2P-50 4E column (Showa Denko, Tokyo, Japan), and the amount of released Gal was determined by HPLC (LC-2000 series; Jasco, Tokyo, Japan) with a Corona charged aerosol detector (ESA Biosciences, now Thermo Fisher Scientific). One unit of enzyme activity was defined as the amount of enzyme that releases 1 μmol of Gal/1 min/1 nmol of enzyme under our experimental conditions.

## Data availability

The structures presented in this paper have all been deposited in the Protein Data Bank (PDB) with the following codes: 7BYS, 7BYT, 7BYV, and 7BYX. All remaining data are contained within the article.
