# Supplementary material for: Unique active-site and subsite features in the arabinogalactan-degrading GH43 exo-β-1,3-galactanase from Phanerochaete chrysosporium
Source: J Biol Chem. 2021 Jan 13;295(52):18539–52. doi: 10.1074/jbc.RA120.016149 (PMC7939473; doi:10.1074/jbc.RA120.016149)
Supplement: Supplementary file 1 [file mmc1.pdf]

Supporting information for

Unique active-site and subsite features in the arabinogalactan-degrading GH43 exo-β-1,3-galactanase from *Phanerochaete chrysosporium*

Kaori Matsuyama[1], Naomi Kishine[2], Zui Fujimoto[2], Naoki Sunagawa[1], Toshihisa Kotake[3], Yoichi Tsumuraya[3], Masahiro Samejima[1,4], Kiyohiko Igarashi[1,5*], and Satoshi Kaneko[6]

1 Department of Biomaterial Sciences, Graduate School of Agricultural and Life Sciences, The University of Tokyo, 1-1-1 Yayoi, Bunkyo-ku, Tokyo 113-8657, Japan

2 Advanced Analysis Center, National Agriculture and Food Research Organization (NARO), 2-1-2 Kannondai, Tsukuba, Ibaraki 305-8518, Japan

3 Department of Biochemistry and Molecular Biology, Faculty of Science, Saitama University, 255 Shimo-Okubo, Sakura-ku, Saitama 338-8570, Japan

4 Faculty of Engineering, Shinshu University, 4-17-1, Wakasato, Nagano 380-8533, Japan

5 VTT Technical Research Centre of Finland, PO Box 1000, Tietotie 2, Espoo FI-02044 VTT, Finland

6 Department of Subtropical Bioscience and Biotechnology, Faculty of Agriculture, University of the Ryukyus, 1 Senbaru, Nishihara, Okinawa, 903-0213, Japan

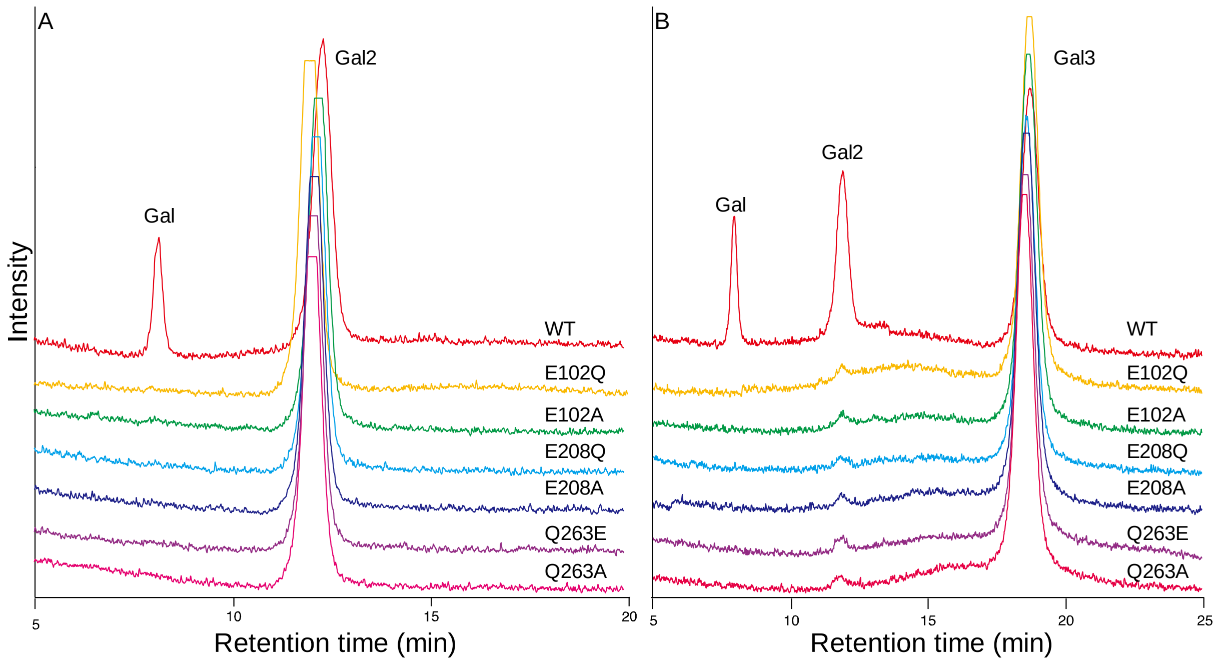

Fig. S1. HPLC analysis of hydrolysis products formed from Gal2 (A) and Gal3 (B) by *Pc*1,3Gal43A_WT and its mutants.

Chromatograms of incubation mixtures of 0.263 mM Gal2 (A) and 0.266 mM Gal3 (B) with WT and each mutant. The enzyme (20 nM) was incubated with each substrate in 20 mM sodium acetate, pH 5.0, for 30 min at 30 °C. The reaction mixtures were subjected to HPLC. The amount of released Gal was calculated based on the peak area.

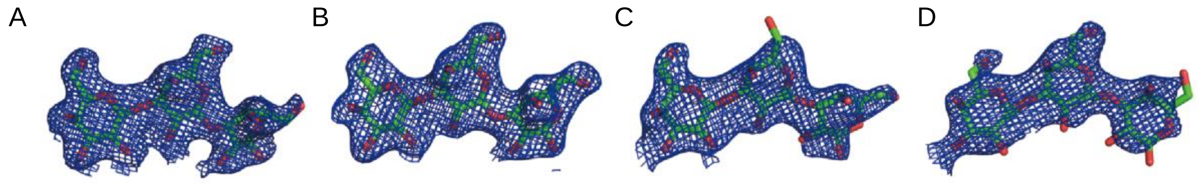

Fig. S2. 2Fo-Fc omit maps (1.0 sigma) of Gal3 at the CBM ligand binding site. A–D show each chain of E208A. The left side is the non-reducing end and the right side is the reducing end.

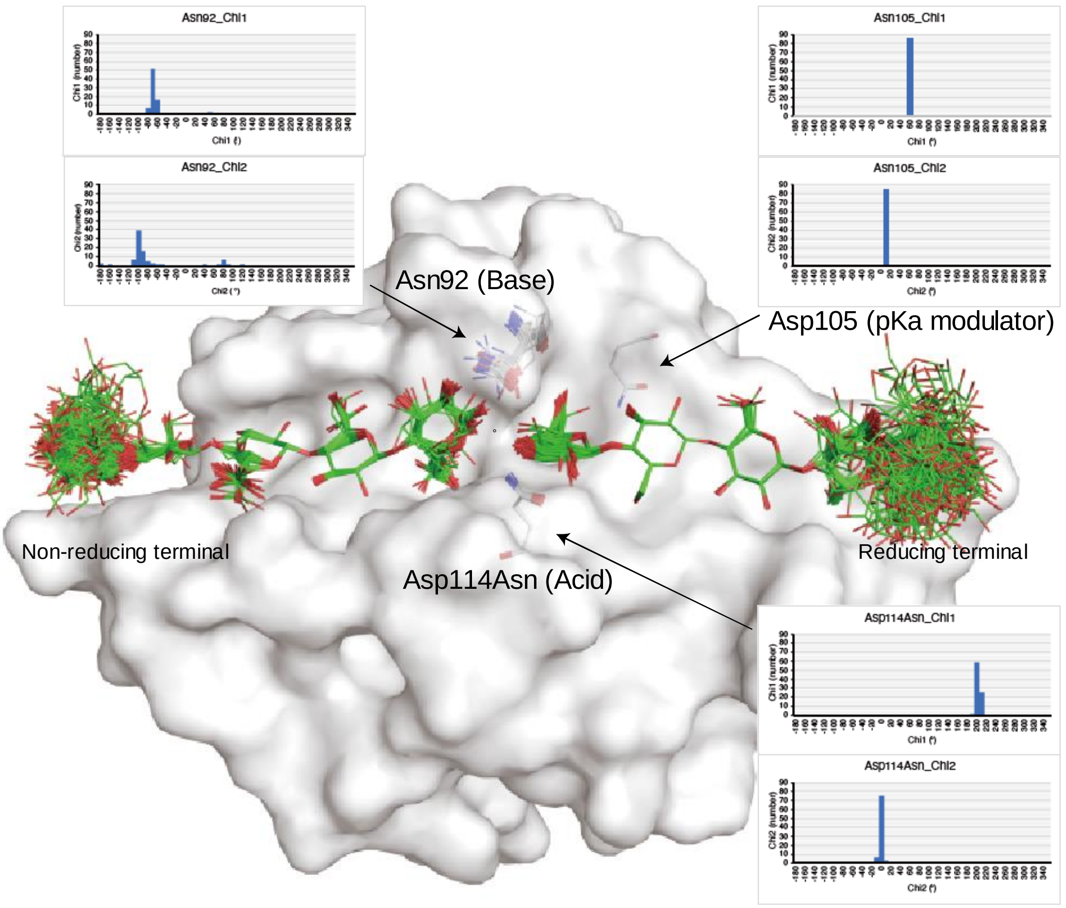

Fig. S3. Catalytic residue fluctuation of *Pc*Cel45A. Ensemble refinement was performed for *Pc*1,3Gal43A with *Pc*Cel45A D114N complexed with cellopentaose (PDB ID: 3X2K). In total, 86 states were calculated, representing all catalytic residues and ligands (two molecules of cellopentaose). Histograms of the dihedral angles of the three residues involved in the catalytic reaction are shown in the diagram.

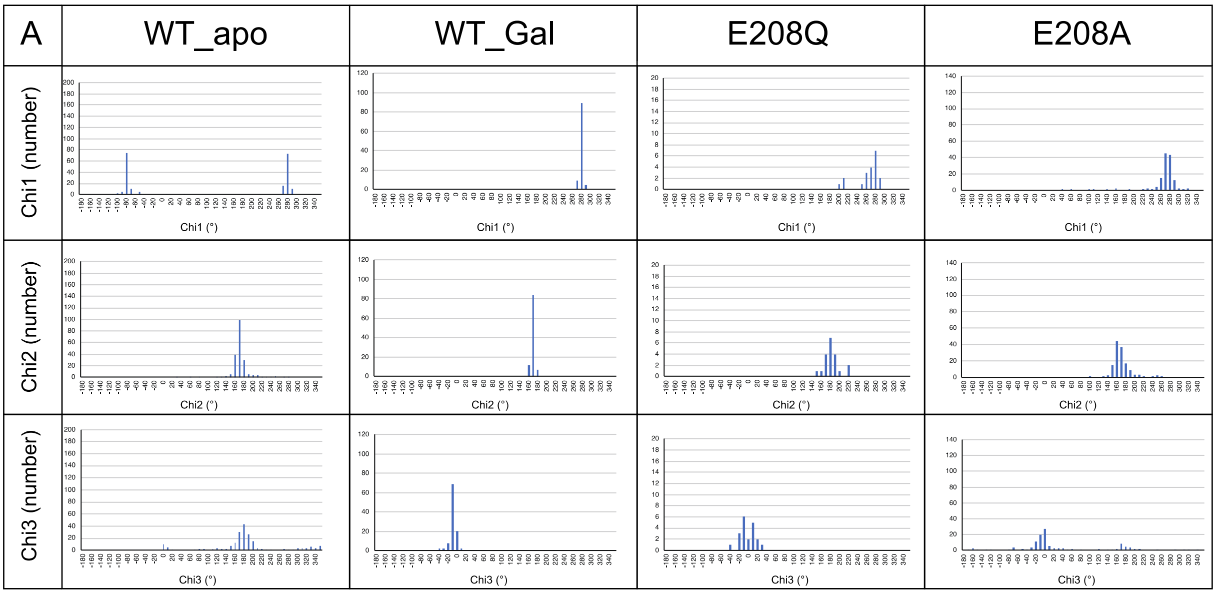
A
WT_apo
WT_Gal
E208Q
E208A
Chi1 (number)
Chi2 (number)
Chi3 (number)
Chi1 (°)
Chi2 (°)
Chi3 (°)

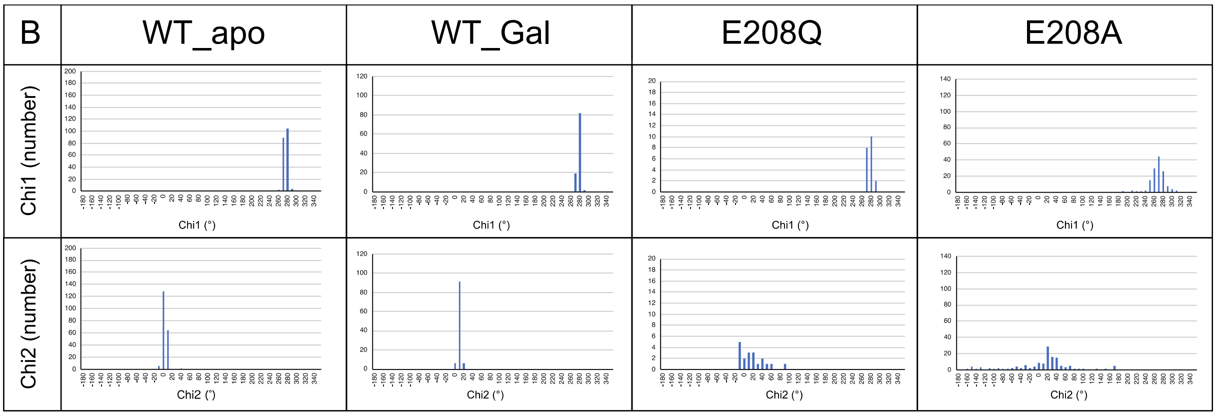
B
WT_apo
WT_Gal
E208Q
E208A
Chi1 (number)
Chi2 (number)
Chi1 (°)
Chi2 (°)

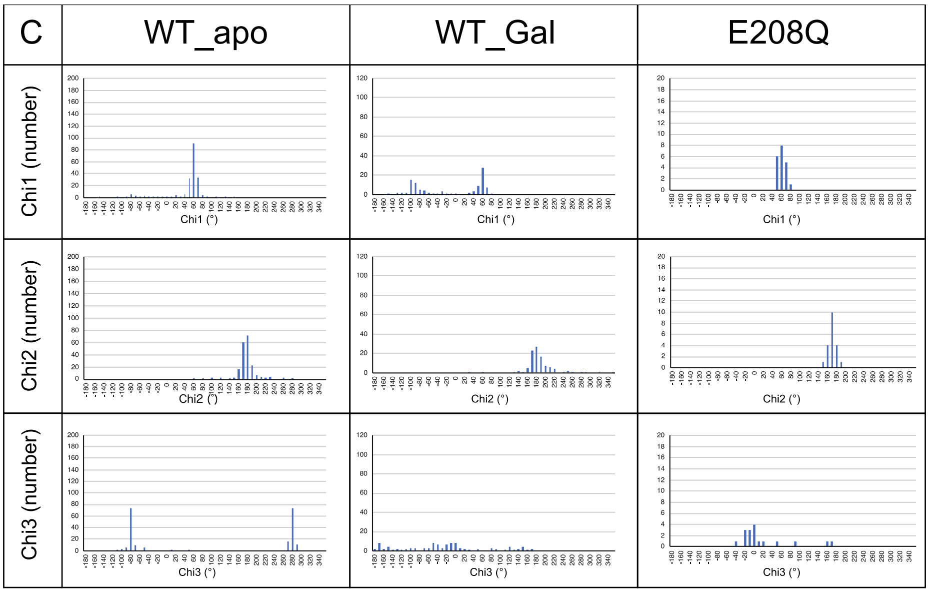
C
WT_apo
WT_Gal
E208Q
Chi1 (number)
Chi2 (number)
Chi3 (number)
Chi1 (°)
Chi2 (°)
Chi3 (°)

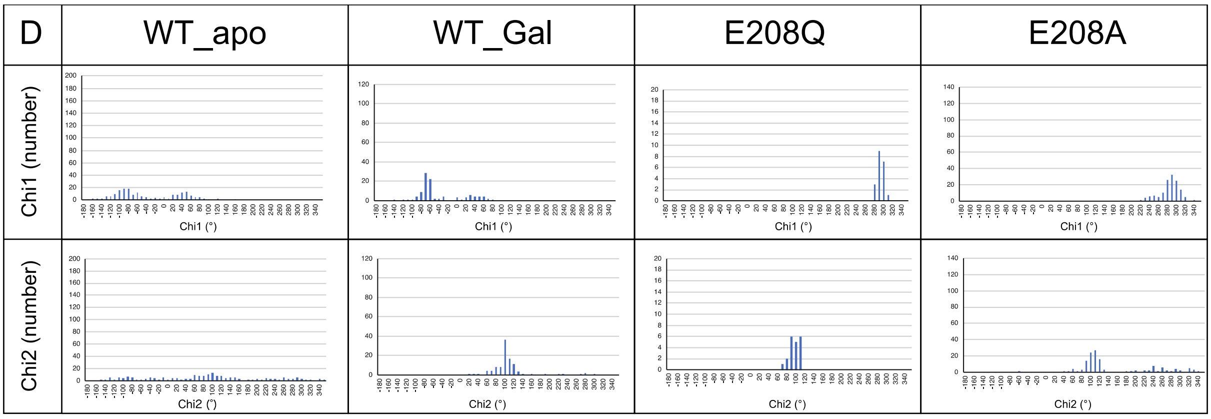
D
WT_apo
WT_Gal
E208Q
E208A
Chi1 (number)
Chi2 (number)
Chi1 (°)
Chi2 (°)

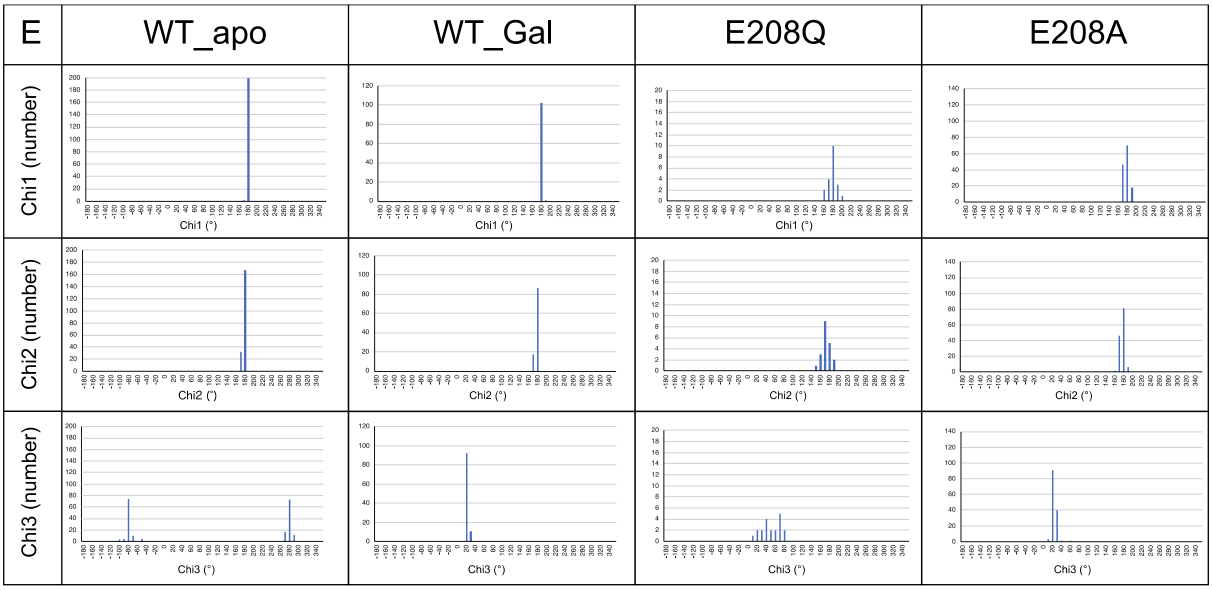
E
WT_apo
WT_Gal
E208Q
E208A
Chi1 (number)
Chi2 (number)
Chi3 (number)
Chi1 (°)
Chi2 (°)
Chi3 (°)

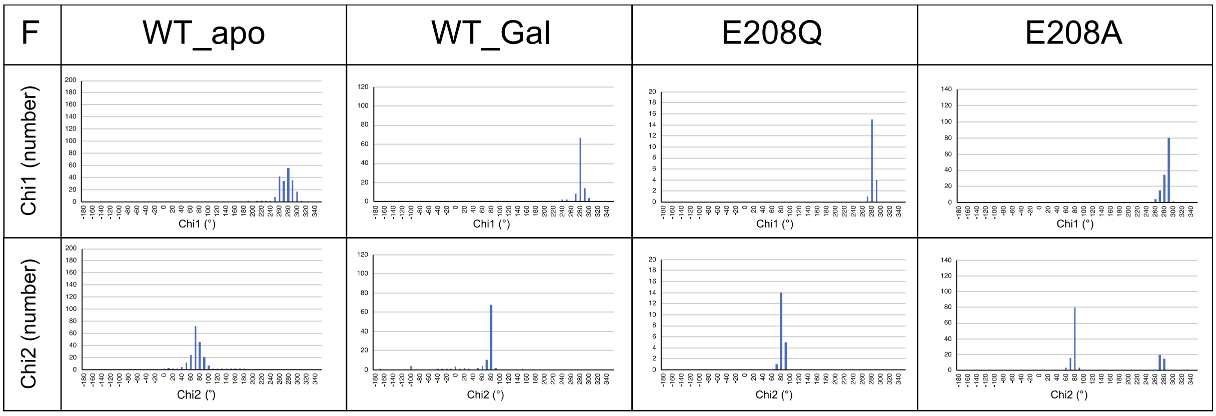
F
WT_apo
WT_Gal
E208Q
E208A
Chi1 (number)
Chi2 (number)
Chi1 (°)
Chi2 (°)

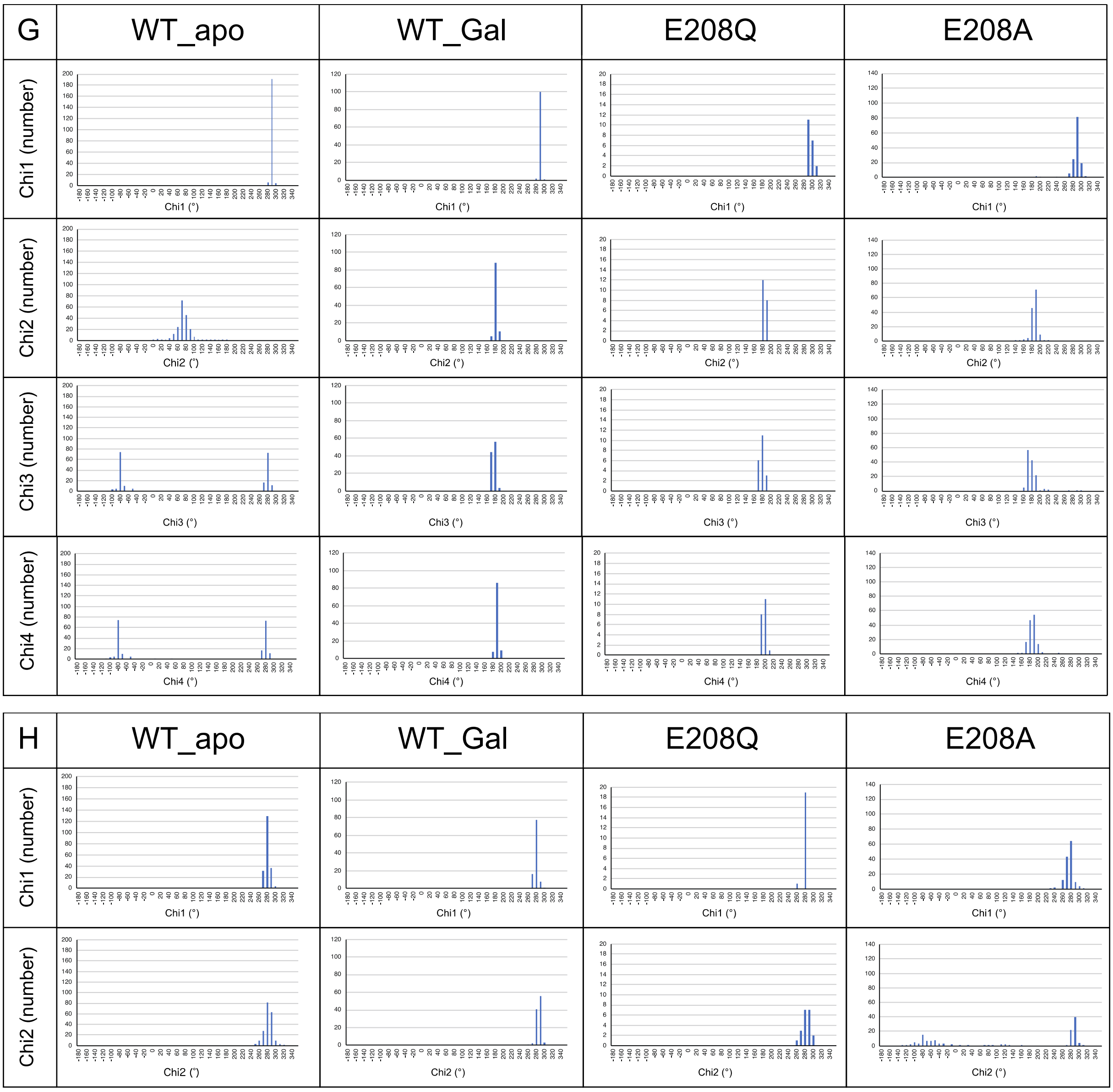

Fig. S4. Histograms of the dihedral angles of each conformation obtained by ensemble refinement of amino acid residues involved in catalysis and substrate recognition. A–H indicate Glu102, Asp158, Glu(Gln)208, Trp229, Gln263, Tyr355, Arg388, and Tyr438, respectively.

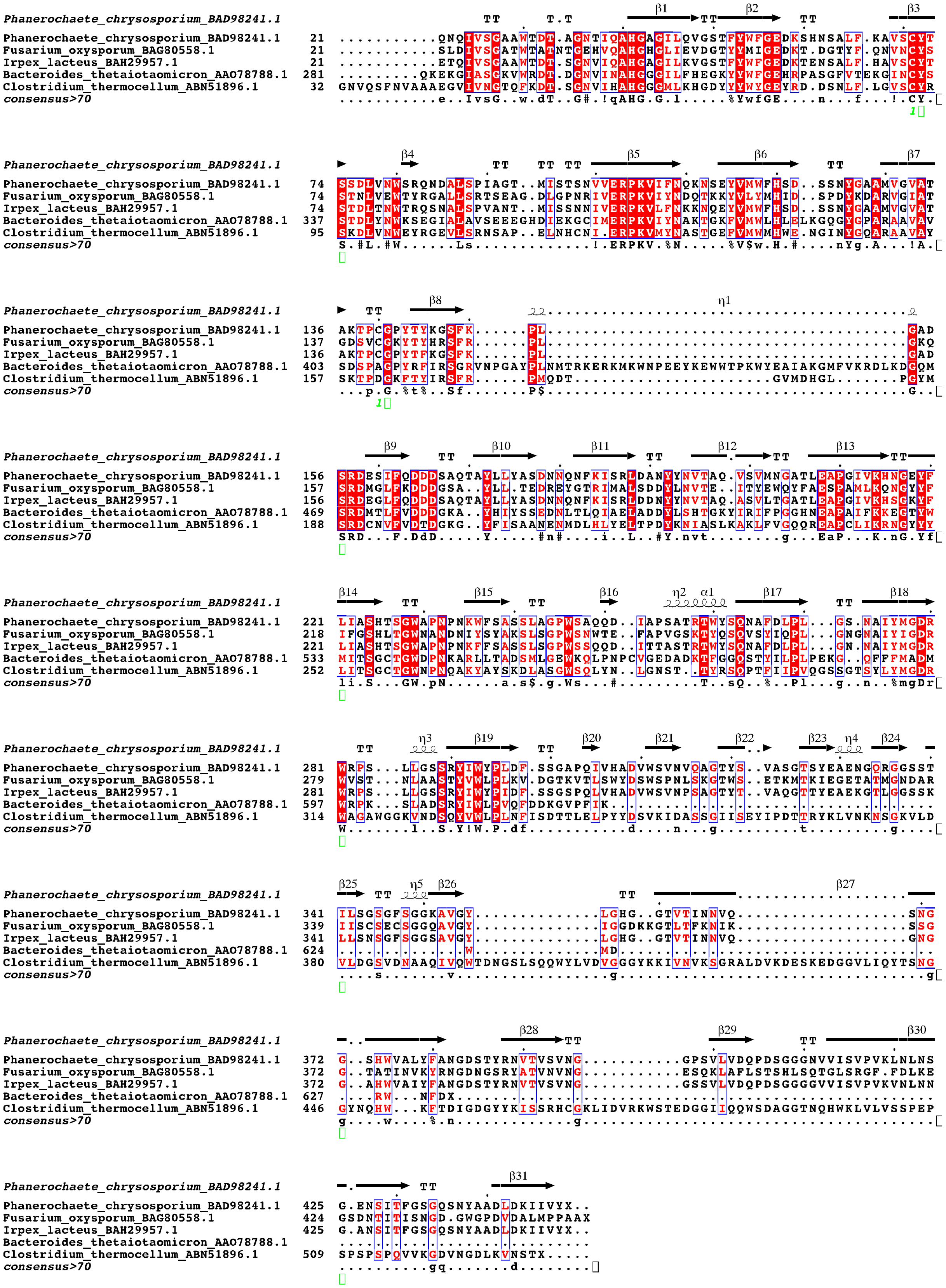
Phanerochaete_chrysosporium_BAD98241.1
Fusarium_oxysporum_BAG80558.1
Irpex_lacteus_BAH29957.1
Bacteroides_thetaiotaomicron_AAO78788.1
Clostridium_thermocellum_ABN51896.1
consensus>70

Figure S5. Sequence alignment of GH43_sub24 galactanases of two basidiomycetes, one mold, and two bacteria. The alignment was built by using MUSCLE on MEGAX: Molecular Evolutionary Genetics Analysis (53, 54), and the figure was generated with ESPrint 3.0 (http://espript.ibcp.fr; 55). The rate of conservation of the GH43_sub24 region (up to approximately the sixth row in this alignment) is high, although C-terminal region is less well conserved because of the differences of the CBM.

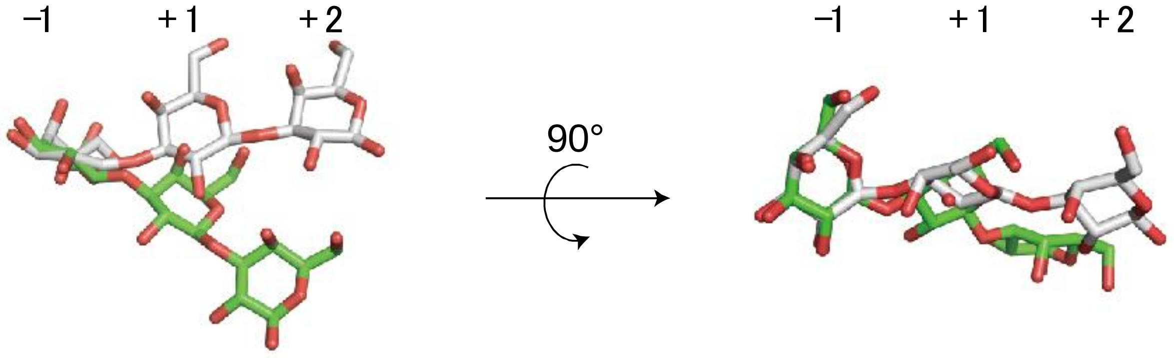

Fig. S6. Comparison of galactan conformation.

Comparison of Gal3 conformations between catalytic-site-bound Gal3 of *Pc*1,3Gal43A_E208Q (white) and the "ideal" conformation of Gal3 (green). The ideal model was built by using web tool "SWEET" (http://www.glycosciences.de/modeling/sweet2/doc/index.php#; 56, 57)
